# Design and Implementation of a Wireless Recorder System for Seismic Noise Array Measurements

**DOI:** 10.3390/s22218103

**Published:** 2022-10-22

**Authors:** Julio Antonio Jornet-Monteverde, Juan José Galiana-Merino, Juan Luis Soler-Llorens

**Affiliations:** 1 Department of Physics, Systems Engineering and Signal Theory, University of Alicante, Crta. San Vicente del Raspeig, s/n, 03080 Alicante, Spain; 2 University Institute of Physics Applied to Sciences and Technologies, University of Alicante, Crta. San Vicente del Raspeig, s/n, 03080 Alicante, Spain; 3 Department of Earth Sciences and Environment, University of Alicante, Crta. San Vicente del Raspeig, s/n, 03080 Alicante, Spain

**Keywords:** wireless sensor networks, Wi-Fi networks, CC3200, node.js, seismic noise, data acquisition, array measurements

## Abstract

In this work, a wireless data acquisition system for seismic noise array measurements is presented. The developed system is composed of a series of nodes and a central server arranged in a point-to-multipoint topology. The nodes consist of a CC3200 microcontroller, an analog-to-digital converter, and a low-noise conditioning circuit designed specifically to register seismic noise, and which is connected to the seismic sensor. As a server, a Raspberry Pi 4B has been used that will receive the samples from the nodes via Wi-Fi and will save them in files. It also incorporates a Web interface developed with JavaScript node.js technology that allows to configure the number of nodes as well as different options, to start and stop the records, and to view in real time the different signals received from the nodes. The system can be deployed anywhere since each of the nodes use independent batteries as a power supply. In addition, it is possible to operate the system remotely if internet connectivity is available. The prototype has been tested in four different locations in the Alicante province (southeast Spain), demonstrating its suitability for seismic noise array measurements.

## 1. Introduction

Soil characterization is crucial to studying and quantifying the behavior of soil in the case of earthquakes and provides insights into the possible associated damages [1,2]. Therefore, these studies are especially necessary in populated areas, not only in areas already built but also in areas of possible future urbanization.

Subsoil properties can be investigated by geotechnical (e.g., downhole, etc.) and geophysical (e.g., seismic, electrical, etc.) techniques. In the case of seismic methods, a seismic source is needed, which may have its origin in ambient vibrations (passive measurements) or in artificial active sources (active measurements). Among all of them, the ones based on ambient vibrations (also known as seismic noise) have become increasingly popular in recent decades, as they are non-invasive and easier to implement, which makes them especially suited for urban areas.

Ambient vibrations present very small amplitude variation, around 10^−10^ to 10^−2^ m/s, and bandwidth between 0.001 and 50 Hz [3]. Their acquisition is carried out by one or more sensors, which usually act as velocity-to-electrical signal transducers, in combination with a data acquisition system. The typical sensors used in these applications are short-period sensors (with a high-pass response, cutoff frequency between 0.2 and 4.5 Hz, and sensitivity between 100 and 400 V/m/s) or broadband sensors (with a flat frequency response from approximately 0.01 to 50 Hz, and a sensitivity between 800 and 2000 V/m/s).

Regarding digitizers or data acquisition systems, these can have one or multiple channels, with typical dynamic ranges between 16 and 24 bits. For seismic noise acquisition, the most commonly used sampling frequency is 100 or 125 Hz [4].

Among the different methods based on seismic noise measurements, one of the most commonly used for soil characterization is the horizontal-to-vertical spectral ratio (H/V) method [5,6,7].

In this case, a single three-component sensor is required. The relationship between the vertical component and the average of the two horizontal components provides information on the resonance frequency of the soil under study [8,9].

If any of the frequencies of the seismic waves coincide with the resonant frequency of the ground, then their amplitude will be amplified, increasing the effects of the associated earthquake.

Array techniques based on seismic noise measurements are also remarkable tools for soil characterization. The only assumptions that have to be fulfilled during each measurement are homogeneous and isotropic soil conditions under the area covered by the array [10] and stochastic and stationary noise wavefields in both the space and time domains [10,11,12].

These techniques require several vertical sensors recording simultaneously. After that, different techniques can be applied for data analysis. Three of the most widely used methods are the spatial autocorrelation (SPAC) technique [10,13,14], the extended spatial autocorrelation (ESAC) method [10,14,15], and the frequency-wavenumber (f-k) method [16,17,18].

**Figure 1:**
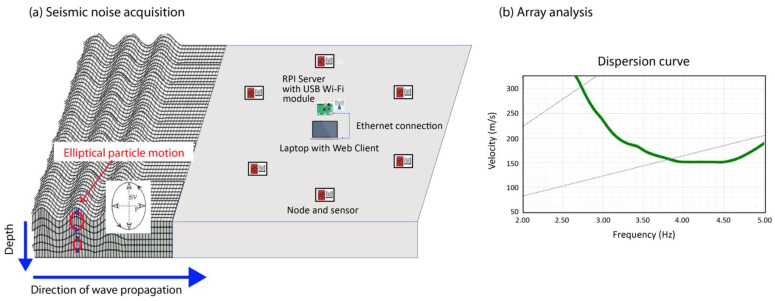
(**a**) General scheme of the seismic noise acquisition and the array deployment (**b**) Example of the result obtained after the analysis, i.e., dispersion curve.

The application of any of these methods allows to obtain the velocity of the propagation of the Rayleigh waves through the medium as a function of frequency, which is known as the dispersion curve (Figure 1).

Once the dispersion curve is obtained, the velocity profile of the corresponding shear-wave (SV in Figure 1) can be estimated through an inversion process, and the type of soil can be characterized, which is of great importance to knowing how an earthquake can affect the study area [11,19,20,21].

From the point of view of its implementation and the necessary resources, all these techniques require several sensors and a multichannel digitizer to record several signals simultaneously. The acquisition of these digitizers, together with the corresponding sensors, represents a significant economic outlay that is not within the reach of all research groups. For this reason, advances in research related to the characterization of the subsoil or the seismic hazard are often limited by the excessive cost of the required instrumentation.

Commercially, there are seismic signal acquisition pieces of equipment with one or three components that can register as autonomous stations, record the data locally in each piece of equipment, and/or transmit them via cable or wirelessly to a central piece of equipment. However, this requires several sensor-acquisition systems, which makes the cost even more expensive. Additionally, above all, it is not in itself an optimized system prepared for array measurements.

There are other commercial pieces of equipment that have multichannel acquisition systems to which several sensors can be connected and whose main purpose is the simultaneous measurement of several geophones or seismic sensors. Most of this equipment is wired, which makes it difficult to implement in certain urban environments, where the layout of the geophones must be adapted to the free spaces available and the corresponding geometries.

All this, together with the growing development of microcontrollers and open-source electronic platforms, has led to the development of ad hoc seismic recorders by some researchers. Many of them are focused on the measurements of a vertical sensor, e.g., [22,23], the measurements of three components for subsequent H/V analysis, e.g., [24,25,26], geophysical exploration, e.g., [27,28], seismic monitoring in buildings, e.g., [29,30,31], etc.

In the specific case of array measurements, the acquisition of the signals from each sensor must be carried out in a synchronized and coordinated manner, acting as a whole and not each one individually.

Picozzi et al. [32] developed a wireless system for seismic noise array measurements. In this case, the signal from the seismic sensor is directly connected to the analog-to-digital converter (ADC), which incorporates an anti-aliasing filter but no other type of signal conditioning. System synchronization as well as temporal stability are associated with GPS accuracy.

In the work of Soler-Llorens et al. [33], a network of wireless sensors (or nodes) is established using the Zigbee communication protocol to manage the acquisition of seismic signals and the synchronization between the measurements of each sensor. The data are collected locally on each node, so the main drawback is that you have to remove the memory cards from each node and access the files one by one. In addition, this system does not allow the visualization of the signals in real time, which prevents problems associated with the malfunction of a geophone or errors in the connections from being detected in situ in the field.

In the work of Yin et al. [34], a system of several arrays of sensors is presented that uses a Wi-Fi connection to communicate between sensors and LTE technology for communication between the different arrays and the seismic workstation. In this case, the object of the work focuses on the field of seismic activity (they used an air-driven, mechanical, artificial seismic source for the experiments), so the signals of interest are registered directly by connecting the sensor to the ADC. However, in the case of passive measurements, considering the amplitude of the ambient vibrations and the typical sensitivity of the sensors, the measured small voltages can usually not be recorded with any recording device directly and must first be amplified [35]. Regarding the synchronization of the system, as well as the temporal stability, they are associated with the precision of the GPS signal. Therefore, as it is commented in this work, a GPS signal interruption can lead to PPS (pulse per second) loss and consequently to an interruption in the acquisition.

In summary, in some of the previously mentioned systems, seismic signals are connected directly to the analog-to-digital converter or to a relatively low signal-to-noise ratio (SNR) acquisition circuit. This makes them suitable for relatively high amplitude signals, but less so for seismic noise.

Regarding the synchronization between stations, in some of the prototypes, it is associated with the accuracy of the GPS signal. In addition, the possible temporal drift due to the internal clock of the microprocessors is not taken into account. For measurements of several hours, this drift can introduce errors in the subsequent analysis of the signals.

Concerning data monitoring, the previously mentioned systems do not allow us to view data in real time. Moreover, in some of the cases, the data recording is performed locally in each of the sensor network nodes.

In the present work, we have designed and implemented a new prototype for array measurements, which overcomes some of the issues mentioned above and satisfies the requirements sought to carry out seismic noise array measurements with low-sensitivity sensors. Below, some of the characteristics that the designed system possesses, unlike those previously referenced, are mentioned:

(1) Low-noise conditioning circuit, which allows using short-period sensors with small sensitivity.
(2) The ADC used allows the acquisition of data from vertical geophones or from three-component sensors so that with the same measurement it will be possible to apply array analysis techniques and the H/V method. In contrast, the ADC1282 used in [34] is a two-channel ADC.
(3) The synchronization between stations is performed via software, which avoids any dependence on an external signal such as GPS. It is important to highlight that a deviation of two or three samples in some stations can make the subsequent array analysis erroneous.
(4) The designed Web user interface allows the remote monitoring of the seismic noise measurements in real time, which allows for verifying that the acquisition is being carried out correctly.

Concretely, the developed equipment is a wireless array seismic noise acquisition system based on Wi-Fi connection and star or point-to-multipoint topology. The sensors are placed around a central server with the characteristic of being able to visualize the received signals in real time and start/stop the measurements through a client browser in a centralized way. The registered signals are saved on the central server almost immediately, without having to extract them individually from each node. Simply by remotely accessing the server, the files can be downloaded to process them.

Some of the bases have been taken from a previous work carried out by the same authors [30]. In both works, point-to-multipoint topology is used. However, in the proposed system, all nodes share the same Wi-Fi network, unlike what happens in [30]. It has led us to implement an exclusive mechanism based on transmission time slots so that each node transmits without causing a collision. Otherwise, all nodes would transmit at the same instants of time since they are synchronized. The types of messages used for communication are very similar to [30], although their structure is different, and some new ones have also been added. Regarding the synchronization, it has been improved, reducing the maximum deviation to a quarter of that achieved in [30]. Finally, the user interface template used in [30] has been used as a starting point for the current one, although it has been improved with additional setup commands. Besides, the data visualization is automatically adapted to show signals from vertical or three-component sensors.

Some of the main novelties and advantages of the developed system are described below:

(1) A low-noise signal conditioning circuit, which adapts the low-amplitude seismic noise to the dynamic range of the analog-to-digital converter (ADC). In this sense, for example, the internal noise level of the selected instrumentation amplifier (i.e., INA128) is twenty times lower than the noise of the amplifiers used in [30,33].
(2) An external 24-bit module (i.e., ADS1256), instead of the internal 12-bit ADC of Arduino (e.g., [33]) or the LaunchPad CC3200 board (e.g., [30]). Thus, the resolution of the system has increased, and the quantification error has been reduced considerably.
(3) The right performance with low-sensitivity geophones for seismic noise measurements. The proposed system has been designed to work with 4.5 Hz geophones, which present low sensitivity (below 100 V/m/s) in comparison with 1 Hz sensors. While the cost of a 4.5 Hz geophone is less than 100 euros, that of a vertical 1 Hz sensor is more than 1000 euros. Therefore, the savings that the use of the proposed system can imply is more than significant.
(4) Each node can be connected to a vertical geophone or a three-component sensor independently. Thus, H/V and array methods can be applied simultaneously to the recorded signals.
(5) A higher transmission rate between the nodes and server through the UDP protocol. This has involved the invention and implementation of a proprietary system that controls the orderly flow of the packets containing the samples and the control of errors and recovery.
(6) Synchronization between all the nodes, and control of the deviation or drift between each one of them and the server. This aspect is essential for the subsequent correct analysis of the recorded signals. No external time source is needed to correct possible deviations (e.g., no GPS is required).
(7) The designed prototype performs from small measurements of short duration (e.g., 5–10 min) to continuous measurements. For long records (e.g., longer than 1 h), it is important to note that if the nodes are not properly synchronized, the recorded signals will become increasingly out of phase, as the record is longer due to the poor quality of the quartz crystal that the microcontrollers contain, which generates a time drift of the internal clock. For this reason, we make a special mention of the synchronization module which is based on the PTP protocol (Precision Time Protocol).
(8) The quick and easy deployment of the entire system to perform the seismic noise array measurements, saving time since no type of wiring has to be deployed to the nodes.
(9) The visualization of each of the signals sampled by each node in real time. In this way, they can detect if it is being measured correctly or if there is a problem with a node. Besides, the system automatically detects if the node has a vertical or a three-component sensor connected and automatically configures the Web interface and plots the panels corresponding to one or three channels at a given node.
(10) The centralization of the sensor samples and the availability of the record in a standard format as soon as the measurements are finished.
(11) The possibility of remote management by connecting the server to the Internet.
(12) The system has been designed to work with up to 10 sensors covering an area of approximately 250 m in diameter, which satisfies the needs required in previous seismic microzonation studies, e.g., [21,36,37,38].

Finally, a series of experiments have been carried out to validate the functionality of the proposed system. The nodes have been proven to be fully synchronized and therefore record the movement of the ground at the same time. In addition, array measurements have also been carried out in four sites of the province of Alicante (southeast of Spain) with the developed system and with commercial equipment. The analysis of the data recorded by both acquisition systems demonstrates the suitability of the designed system for taking seismic noise array measurements.

## 2. System Development and Implementation

### 2.1. Model Description

The designed system follows the client–server concept. The clients are implemented using a Texas Instruments (TI) CC3200 microcontroller [39] (nodes) and the server is configured through a Raspberry Pi computer [40] (RPI server). The system consists of several nodes located in a certain area forming a star topology where the RPI server is located in the center. Each node incorporates a specially designed expansion board with a new conditioning circuit, which is connected to a one-component seismic sensor. The RPI server incorporates a Wi-Fi antenna with a 5 dBi gain (model BrosTrend AC650) [41] that acts as an Access Point (AP) and provides the Wi-Fi signal to the nodes. These communicate with the server via a UDP (User Datagram Protocol). A JavaScript (JS) server has been developed in the RPI server that manages the communication through UDP connections and receives the samples from each node. In addition, the received data are saved in local files and displayed through a Web user interface in real time

Figure 1a shows the diagram of the developed system. A star topology is used, where the RPI server is placed in the center and the nodes are distributed around it. From the Web interface, you can configure the number of nodes that will be registering the signal (up to a maximum of 10 nodes),

It has been decided to use UDP as the transport protocol as it provides a higher transmission rate than TCP (transmission control protocol). According to the CC3200 Launchpad [39] specifications, the rate for UDP is 16 Mbps, and for TCP, it is 13 Mbps. In addition, it must be taken into account that the deployed network is a dedicated network for the nodes and therefore there will not be any more hosts. It follows that there will be no congestion on that network, so it does not make much sense to use TCP. More comparative details between TCP and UDP over wireless networks can be found in the work of Xylomenos and Polyzos [42].

As UDP is not a connection-oriented protocol, the application layer must be in charge of controlling and managing the delivery of the packets in order to avoid their loss. Therefore, some procedures have been implemented to minimize the loss of frames containing samples. A recovery mechanism has been also implemented in case of the loss of the Wi-Fi connection during the sampling process.

In addition, a new signal conditioning circuit has been designed to amplify and filter the signal obtained from the seismic sensor, in such a way that it is adapted to the dynamic range of the ADC. The output signal is sampled with an external 24-bit ADC module (ADS1256) [43] that communicates with the CC3200 board via the SPI (serial peripheral interface) bus. The sampling frequency of the ADC is 100 Hz, which perfectly captures the frequencies of interest associated with the ground vibrations, i.e., seismic noise.

The nodes can be configured to sample one or three components, depending on the type of sensors, by means of a jumper. Thus, the deployed array can include one- and three-channel sensors at the same time, which allows for applying HVSR and array techniques with the same measurement recordings. The Web server configures itself and displays the graph of one or three channels for each sensor.

A minimum of 3 bytes (24 bits) is required to record each of these data. In addition, for synchronization purposes, a millisecond mark is also transmitted with the data. Thus, the minimum payload that the protocol must assume is 8.8 Kbps, which corresponds to a constant rate of 100 samples × 3 values × 24 bits and 100 samples × 16 bits (milliseconds mark). To be as efficient as possible, a single Ethernet frame is used to transmit a block of samples. The maximum size of the Ethernet frame is 1518 bytes, so we will transmit blocks of 100 samples (1100 bytes and 16 bytes of header) every second, as two seconds would exceed the maximum size and we will have to use segmentation.

### 2.2. Raspberry Pi Server

The server has been developed on a Raspberry Pi 4 Model B 4G [40] with Raspbian GNU/Linux 10 core 5.10.63. This model incorporates a Wi-Fi interface, but its coverage (about 10 m according to the test carried out) is not enough for our purposes. In addition, there is no possibility of increasing the coverage by adding an antenna. Therefore, the solution adopted has been to use a second USB Wi-Fi interface (i.e., BrosTrend AC650) [41]. With this configuration, it is possible to connect the RPI to the internet and thus be able to manage the Web.

This computer has been chosen because one of the objectives is to design a portable, low-cost system that allows us to easily perform soil measurements in an array configuration. If at the time of taking the measurements, the RPI server does not have an internet connection, then you must have a laptop connected to the RPI server to be able to start the measurements locally. If a connection to the internet were available, then a local presence would not be required, and measurements could be initiated from anywhere.

To manage the connectivity of the nodes, the RaspAP package has been installed, which facilitates the configuration of the interfaces and the AP. With RaspAP, you can choose which Wi-Fi channel to use, which is important to avoid any interference with existing channels in the area. Thus, prior to the deployment of the system, a scan of the medium is necessary to know the occupied channels. In addition, the Node.js [44] package has been installed in order to provide the Web service and execute the JavaScript code of the server. The main features implemented in the code are:

- UDP connection management.
- The management of received packets and the storage of samples.
- Synchronization management.

The RPI server has been configured in NTP (network time protocol) client mode so that it automatically synchronizes its clock with a master clock, and in turn, also acts as an NTP server to provide the clock reference to the nodes. In this way, the nodes will be synchronized when roughly booting to the RPI server for the first time. The SCP (secure copy protocol) service has also been installed to access the sample files and download them.

#### 2.2.1. UDP Connections

For communication between the nodes and the RPI server, two sockets have been created so that communication is full-duplex. The RPI server will be listening to UDP port 8000 and the nodes will be listening to UDP Port 8001. It is a point-to-multipoint communication. When the Node.js server is executed in the RPI server, immediately, port 8000 is opened to receive frames from the nodes, and TCP port 8080 is opened to receive Web client requests from a browser. As the nodes start up, they open port 8001 and send a Hello frame to port 8000 of the RPI server, indicating the number of nodes and the number of configured acquisition channels (corresponding to one- or three-component sensors). From this moment on, each node will send a Hello frame every 10 s. As the RPI server receives the Hello frames, it will save the IP of each node and its corresponding number of channels.

A timeout has been activated in each connection to detect the shutdown of a node. Concretely, the timeout has been set up to 32 s, which corresponds to the loss of three consecutive Hello messages. Two seconds have been added to the timeout for considering any possible delay in the local network due to increased traffic or any other cause.

Twenty seconds after starting the node.js server in the RPI server, the synchronization of each node with the NTP server will start.

Once all the nodes have been synchronized and when the recording begins, practically all the nodes will sample in unison and therefore will capture the 100 samples at the same time. At this moment, each node will generate the frame and send it via Wi-Fi. This situation will generate collisions in the Wi-Fi medium since all the nodes will try to transmit at the same time. To avoid this situation, time slots have been established so that each node will have its moment to transmit.

Thus, the maximum number of nodes supported by the system will be determined by the total number of time slots in which each second is divided. In our case, the system has been designed with 10 time slots, which implies a maximum number of nodes of 10. In this way, each node will have 100 ms of time to send its current frame and also those that are pending to be forwarded (with a maximum of five) if an error has occurred.

The easiest way to implement the delivery order is based on the node number that each device has. So, for node 1, the first frame sent will have only 10 samples (1–10 samples), the second will have 100 samples (11–110 samples), the third frame will contain from 111 to 210 samples, and so on sequentially. Node 2 will send its first frame with only 20 samples, and the successive ones will contain 100 samples: 1–20, 21–120, 121–221, etc. Thus, node 1 will start to transmit at time t_0 + 100 ms, node 2 at time t_0 + 200 ms, node 3 at time t_0 + 300 ms, etc. Therefore, each node will have a 100 ms slot.

In order to guarantee the orderly delivery of the frames and minimize their loss, since UDP lacks these characteristics, an ACK mechanism has been implemented with a sliding window of 10 frames. Its operation is as follows: when in Sampling Mode, each node will send a frame with 100 samples every second and will store them in a buffer. When the RPI server has received the 10 frames in sequential order, it will then send a frame Samples-ACK confirming to the node that it has received the 10 frames correctly. At that moment, the node will erase the 10 frames and will continue with this process. At all times, the RPI server keeps track of the sequence number it expects to receive; if it does not match because the sequence it receives is greater than expected, it will mean that one or more frames have been lost, and at that moment, it will send a frame Samples-ACK to the node in question with the last sequence number received correctly. The node, upon receiving the ACK, will detect that it must resend one or more frames, depending on which ones have been lost.

In the case of having to retransmit one or more frames, the node will have 100 ms to transmit them. A maximum number of frames to be transmitted is defined to guarantee that they do not overlap with those of the next node, and that is six frames. If the block to retransmit is greater than six, it will have to be performed in several time slots until all the lost frames have been retransmitted.

A buffer has been defined to contain up to 100 frames (maximum), which is equivalent to 100 s. Thus, the system is capable of operating without losing samples with a communication cut-off of up to 100 s. After 100 s, the frames with samples will begin to be lost and the RPI server will detect the lost frames.

#### 2.2.2. Packets Management and Sample Recording

Regarding the connection between the RPI server and the Web client, and the exchange of information between them, the WebSockets library [45] has been used. The samples received by the RPI server are sent to the Web client to plot them in the graph using this library. If the RPI server has a Web connection, then it will send to the client browser 1 sample out of 10 received by the nodes to be displayed in a chart using the Highcharts [46] for JavaScript object. This is performed so as not to saturate the browser and because such precision is not required at the time of measurements. If the SAVEDATA option has been activated in the FrontEnd, the samples will be saved in a specific directory of the RPI server. The samples will be saved every 15 min in files that the code itself will generate.

### 2.3. Nodes: Conditioning Circuit and Analog-to-Digital Conversion

Based on the above considerations, the voltage signal provided by the geophone in the case of ambient noise measurements is of very low amplitude, and its direct connection to the analog-to-digital converter would only provide a very low-resolution signal and would not be useful for soil-type characterization purposes.

For this reason, a conditioning circuit is necessary that transforms the signal recorded by the geophone to a range of voltage levels and frequencies that adjust to the dynamic range of the analog-to-digital converter used.

However, given the low amplitude of the recorded signals, simple signal amplification and filtering may not be sufficient. It is important that the internal noise of the designed circuit is below the minimum levels of seismic noise that can be recorded. Otherwise, the signal of interest would be masked by the noise of the circuit itself.

**Figure 2:**
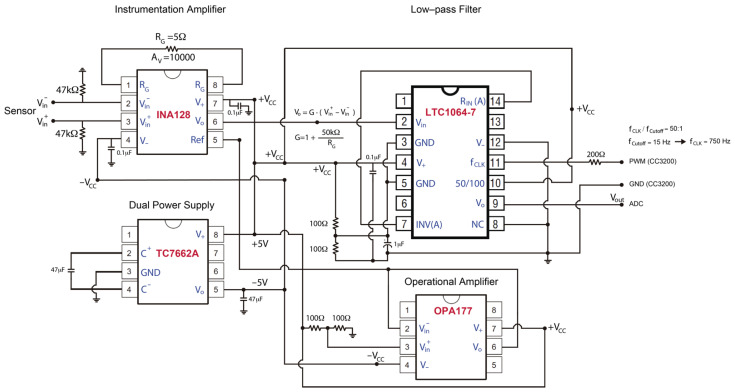
Electronic schematic of the signal conditioning circuit.

In the present work, a low internal noise signal conditioning circuit has been designed and developed. For that, low-noise off-the-shelf components have been selected. In Figure 2, the electronic scheme of the signal conditioning circuit is shown.

The conditioning circuit basically consists of the following modules:

1. Voltage conversion module (TC7662A) [47]. This provides the necessary supply voltage for each of the integrated circuits. It is capable of providing symmetrical voltage. In this case, the integrated circuits work with voltages of 5 V and ±5 V.
2. Instrumentation amplifier (INA128) [48]. This converts the differential signal from the geophone into an amplified signal referenced to the ground. The system gain is controlled by an external resistor connected between pins 1 and 8. Given the low amplitude of seismic noise, the resistor selected is such that it is capable of providing a gain of 1000 or 10,000. The choice of gain depends on the selected site type. On relatively quiet sites, a gain of 10,000 may be more appropriate. In urban places, with a higher level of ambient noise, a gain of 1000 is recommended, since a gain of 10,000 can sometimes saturate the signal. As previously mentioned, the amplitude of the internal noise of the circuit in general, and of the integrated circuits in particular, is crucial for the correct operation of the system. In this case, the INA128 is characterized by a low noise of the order of 0.2 μVpp. The reference signal, connected to pin 5, is set to half the maximum supply voltage; that is, 2.5 V. To do this, a low-noise operational amplifier (OPA177) [49] is used, which acts as a voltage follower with a very low output impedance.
3. Anti-aliasing filter (LTC1064-7) [50]. This is a low-pass filter of order 8, with switched capacitances whose cutoff frequency is controlled by an external clock signal. The square clock signal is provided by the microcontroller itself (CC3200) [39], and in this way, via software, the desired cutoff frequency is chosen. In the proposed system, the sampling frequency is 100 Hz, and the cutoff frequency of the filter has been selected as 15 Hz. This cutoff frequency ensures sufficient attenuation at the Nyquist frequency (avoiding possible aliasing) and at the same time includes the range of frequencies of interest in soil characterization studies.

As for the analog-to-digital converter, the ADS1256 [43] module is used, which allows the digitization of up to eight different channels. In our case, the system has been prepared to register one or three channels, corresponding to vertical or three-component seismic sensors, respectively.

The communication between the analog-to-digital converter (ADS1256) [43] and the microcontroller (CC3200) [39] is carried out through the SPI communication bus. Through the SPI interface, the digitized data are sent to the microcontroller to be saved and transmitted to the server.

It is important to note that the conditioning circuit and the analog-to-digital converter are powered by their own power supply or battery, independent of the one used to power the CC3200 microcontroller. This prevents possible interference that could be propagated between the two systems through the power connections.

**Figure 3:**
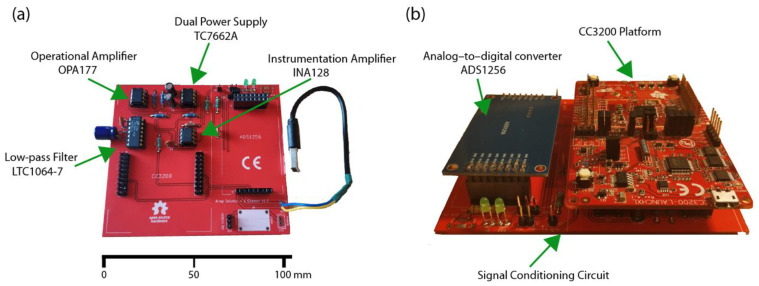
(**a**) Prototype of the signal conditioning circuit in PCB (**b**) Assembly of the complete system, with the conditioning circuit, the ADS1256, and the Launchpad CC3200.

Figure 3 shows the prototype of the conditioning circuit in a PCB (Printed Circuit Board) and the complete hardware system (with the ADS1256 module and the LaunchPad CC3200). The prototype shown is for one channel, and its dimensions are 94 mm high × 115 mm wide.

### 2.4. Nodes: Microcontroller Programming

**Figure 4:**
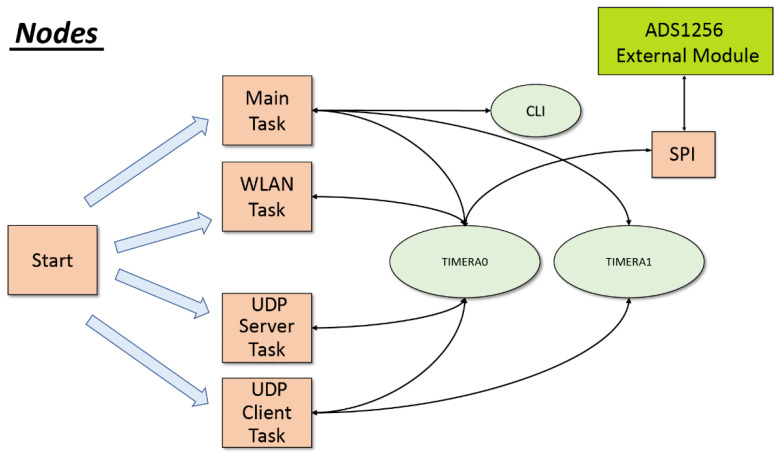
Block diagram of the nodes software. The main functions (square blocks) and interrupts (oval blocks) are shown and their relationship.

Regarding the nodes, the TI LaunchPad CC3200 platform [39] has been selected due to its low power consumption and because the Wi-Fi interface and its libraries are integrated into it. The main software blocks are shown in Figure 4.

Three interrupts have been enabled:

1. TIMER_A0. This is the sampling timer and occurs every 10 milliseconds to capture the samples provided by the ADS1256 module [43].
2. TIMER_A1. This is the main timer of the program and always occurs every one second. It is the heartbeat timer.
3. CLI (Command Line Interface). This is activated every time the UART0 receives a character. This interrupt is used to receive user commands through the UART0 and enable logging.

In addition, four tasks have been created:

1. Main task. In this task, the main functions and routines are executed. Initially, the configuration parameters are read from flash memory. After that, the task enters an infinite loop, where all the associated messages are handled. It is also in charge of starting and stopping the sampling process, setting the timers to be synchronized with the NTP server, and opening and closing the CLI command line session.
2. WLAN task. This handles the functionalities associated with the Wi-Fi connectivity, the time acquisition with the NTP server, and the calculation of the delay and time drift with respect to the RPI server.
3. UDP server task. This task is in charge of receiving the frames coming from the RPI server and listening to the UDP port 8001. It also influences the control of retransmissions since it receives the ACKs from the RPI server.
4. UDP Client task. This task is responsible for managing the socket that communicates with the RPI server (8000 UDP) and sending the packets. It is in charge of constructing the frames defined in our protocol of the application layer and sending them to the RPI server, including the frame with the samples that have just been collected. It is also in charge of controlling the retransmission and the buffer.

When the nodes start up, they are in charge of initializing the ADS1256 module and configuring the number of channels to use.

#### 2.4.1. Sampling Mode

When a node receives a START-SAMPLING packet, the Timer_A0 is activated and triggers every 10 ms. In the interrupt routine, the first thing that is undertaken is to read the value in milliseconds of the TimeStamp (i.e., MilliTimeStamp, mTS) carried by the node through the Timer_A1, and then the values of the one or three channels are read from the ACS1256 through the SPI bus at a speed of 1.9 Mhz. These four values (mTS, Ch1, Ch2, and Ch3) are stored in a buffer, and the counters in charge of controlling the number of total samples (samplesTotalNumber) and the number of samples per packet (samplesPacketNumber) are incremented. When 100 samples are taken, that is, every second, the MSG_SEND_PKT_UDP event is sent to the ClientTask to generate the packet with the 100 values of mTS, Ch1, Ch2, and Ch3, and send it to the RPI server. The inclusion of the mTS information in each packet helps to verify that the sample rate remains stable throughout the time period. The sampling routine spends 4 ns in the data acquisition. It is very important to control this time so that the node has execution time for other tasks.

#### 2.4.2. CLI Interface

A library has been developed with a series of commands that allow for displaying the status of each node and the value of the variables used. In this way, through some of these commands, it is possible to set up the Wi-Fi parameters (SSID and password parameters) and the number of the selected node.

### 2.5. Wi-Fi–UDP Communication

As previously mentioned, three tasks have been created in the nodes to manage communication: the WLAN task, the UDP server task, and the UDP client task. Libraries provided by Texas Instruments have been used in all three tasks.

**Figure 5:**
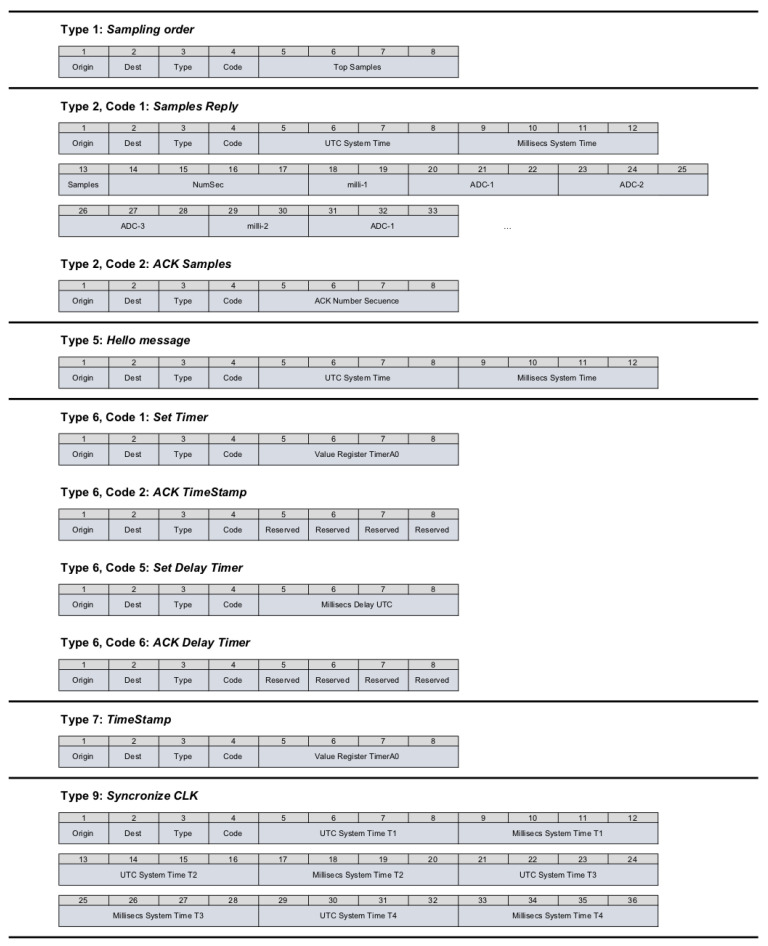
Structure of the messages.

For our application layer, different messages (Table 1) have been defined in order to guarantee functionality with the UDP. The structure of these messages is shown in Figure 5.

Type 1 messages (SO, Sampling Order) are used to send the START/STOP orders of the registers according to the value of the code field:

- Code = 1: Indicates that the node will start sampling immediately after receiving this frame and will send the samples to the server.
- Code = 2: Indicates that the node will end the sampling immediately after receiving this frame and will send the samples that are pending.
- Code = 3: Indicates that the node will start sampling immediately after receiving this frame and will not send any sample to the server.
- Code = 5: Indicates that the node will start sampling in the next second after receiving this frame and will send the samples to the server.

Type 2 messages (SR, Samples Reply) are related to sending and receiving samples.

- Code = 1: Contains the information corresponding to 100 samples. That is a time mark (in milliseconds) that indicates when the samples were taken (2 Bytes); the value of channel 1 of the ADC, which corresponds to the X-axis (3 Bytes); the value of channel 2 of the ADC, corresponding to the Y-axis (3 Bytes); and finally, the value of channel 3 of the ADC, corresponding to the Z-axis (3 Bytes).

In this way, the developed system is prepared for taking array measurements using both vertical and three-component sensors.

- Code = 2: Confirms to the node the correct reception of the samples up to the indicated sequence number.

The Type 5 message corresponds to the Hello frame where the value of the UTC time (universal time coordinated) is stored in seconds (in the UTC System Time field) and milliseconds (in the Millisecs System Time field).

Type 6 messages (ST, Set Timer) are related to setting the time and clock of the nodes.

- Code = 1: Configures and establishes the value of the timer counter that controls the sampling time, which is 10 ms. With this field, we control the time drift of the internal clock of the CC3200 microcontroller.
- Code = 2: Confirms that the node has configured the new value in the timer register.
- Code = 5: Indicates the value in milliseconds of the delay/advance that has to be applied in the internal clock of the microcontroller.
- Code = 6: Confirms that the node has applied the delay/advance to the clock value.

Type 7 messages (TS, TimeStamp) are used to mark the time of both the start of sampling (the time when the first sample of the record is going to occur) and the end of the last sample of the record in each node. These frames serve as marks, calculating exactly the time spent in the register and estimating if the clock of any node needs to be adjusted.

- Code = 1: Indicates the TimeStamp of the start of the record when the first sample occurs.
- Code = 2: Indicates to the RPI server that the node has to be resynchronized and the time drift corrected.
- Code = 3: Indicates the TimeStamp of the end of the record when the last sample occurred.

Type 9 messages (SC, Sync CLK) are used in the synchronization phase for the calculation of the offset and drift.

- Code = 1: The RPI server starts the frame, marking its time.
- Code = 2: The node returns the frame, marking the time when it was received from the RPI server, and also marking the time when it is going to send it back to the RPI server.
- Code = 3: The RPI server returns the complete frame with the four times to the node so that it can calculate the offset and drift.

### 2.6. Synchronization Process

The synchronization process is similar to that developed in paper [30] and consists of two phases. In the first phase, a first-time adjustment is made, and the offset and drift between the node and the RPI server are determined. In the second phase, it is a matter of adjusting the node’s time with respect to the RPI server as much as possible. This synchronization process is inspired by the Time Protocol (PTP) [51] and the Doze Mechanism [52]. For these types of records, the time drift that each node has is not very decisive since the time of the records is usually 15 or 30 min. During this time, the drift is hardly noticeable. The messages used in the synchronization process are Type 9 in the first phase and Type 6 in the second phase.

In the first phase, a sequence of 40 Type 9 messages is sent between a specific node and the RPI server where the TimeStamps are stored. Once received, the offset and drift corresponding to that node are calculated. The estimated offset will be the shortest time that has occurred in the 40 sequences.

In the second phase, Type 5 and 6 messages are used. Every 10 s, the node will send a Hello frame (Type 5) and in the TS field, it will put, just before sending the frame, the TimeStamp that it has in that instant. The RPI server will receive the Hello messages, and when it has received a sequence of 10 Hellos, it will calculate the average of the time difference between the node and the RPI server. In this way, a precise offset will be obtained. When the offset exceeds 5 ms, it will send the Set Delay Timer frame (Type 6, Code 5) indicating to the node when it has to adjust its clock. The node in turn will reply with an ACK (Type 6, Code 6) to confirm the setting to the RPI server.

In this second phase, the Hello messages will be sent sequentially in the second corresponding to the node number. Thus, node 1 will send the Hello when the second’s value will be 1, node 2 at time 2, etc. The next series will occur at times 11, 12, etc. Thus, the RPI server will receive the Hello message corresponding to a given node every second.

**Figure 6:**
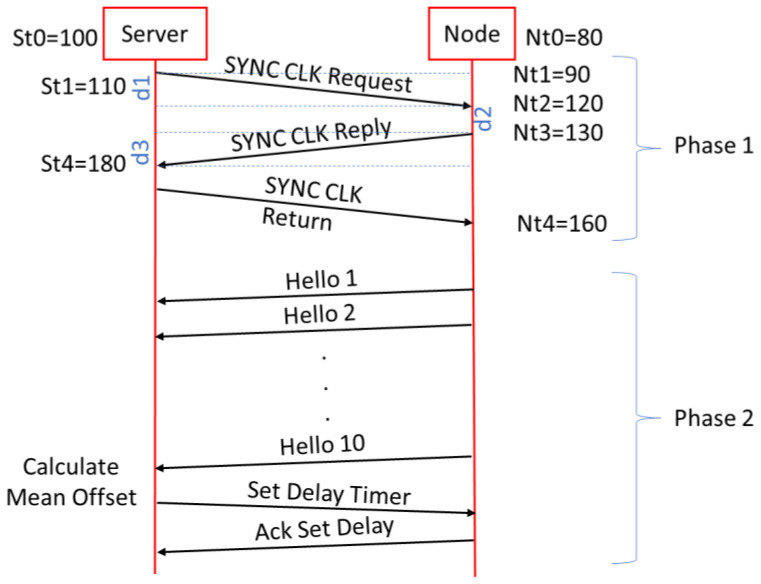
Sequence of synchronization between the RPI server and one node.

The final offset and final time drift respecting the RPI server time are calculated by Equation (1), the meaning of whose variables are in Figure 6. For the example shown in Figure 6, it gives us values of delay T = 60 ms and drift = −20 ms, which means that the node has a delay of 20 ms so it will have to advance its timer by about 20 ms.

(1) $$\begin{array}{l} T_{i}=d_{1}+d_{2}+d_{3}\\ offset_{i}=\left(St_{4}-St_{1}\right)-\left(Nt_{3}-Nt_{2}\right)\\ drift_{i}=\left(Nt_{3}+\frac{d_{T}}{2}\right)-St_{4}\\ offset_{Total}=min\left(offset_{i}\right)\\ drift_{Total}=\frac{1}{20}\overset{20}{\underset{i=1}{\sum }}drift_{i} \end{array}$$

The congestion scenario is not contemplated since the Wi-Fi network deployed will be exclusively for the nodes.

To control the drift and more accurately, adjust the clock of each node; there is the possibility of configuring the counter value that triggers the 10 ms sampling timer. Since external conditions (temperature, humidity) affect the quartz crystal that each node has, a preliminary calibration can be performed for up to 24 h, and at the end, the calculated value that should be configured in the counter of each node will be provided. To configure this value, the Type 6, Code 1 and the Type 6, Code 2 messages will be used.

In addition to the counter setting that provides the fine-tuning of the sampling frequency, the synchro mechanism [30] (p. 15) has been implemented, which automatically adjusts the sampling time when it is being sampled (sampling mode) and can be activated or deactivated from the Web client.

### 2.7. User Interface of the Web Client

**Figure 7:**
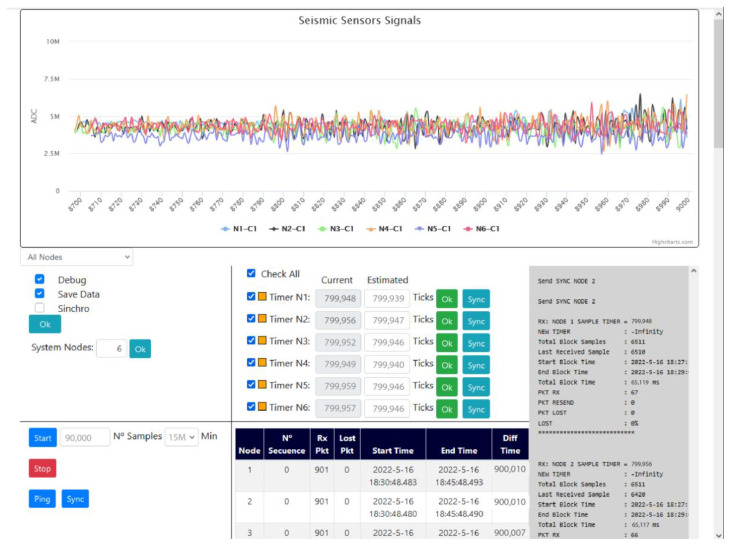
User interface of the Web client.

The Web client part (Figure 7) has been developed with JavaScript language. It consists of a panel where the current signal is displayed, taking into account that only one of every 10 samples received is displayed so as not to saturate the RPI server or the Web client. The purpose is to display an approximation of the received signal. The RPI server automatically detects the channels that each node has, numbering and assigning these components to the graph. We can select to display only the signal of a particular node or a particular component.

Another panel is the Options panel where we find the Debug, SaveData, and Synchro options:

- The Debug option creates and saves in a file the debug messages in the ASCII format for later analysis in case of failures.
- The SaveData option has been developed to save the received samples in files within the RPI server to be able to process and analyze them later. These files are automatically saved with the name “SAMPLES_DATE_TIME” where DATE is the date and TIME is the time of the creation of the new file. A file will be generated every 15 min due to the size of the files. The content of the files is exactly the messages of Type = 2 in the binary format to reduce the file size.
- The Synchro option has already been explained above (Section 2.7).

From the web page, we can configure the number of nodes, and the program will automatically generate the objects to represent all the nodes. In addition, in case of the failure of any of them, or in case it runs out of battery, we can select those nodes that we want to be part of the record to be created. For example, we can have 10 nodes, and node 2 has run out of battery, so then we can cancel it from the register without interfering with the sequence of receiving the samples.

A panel has also been designed to display a table with the important data related to the current sampling block such as the start of the sampling, the number of packets received, etc. Additionally, on the right side of the screen, a panel has been implemented to display important system messages and configuration data, as well as the statistics at the end of a sampling block.

## 3. Results and Discussions

### 3.1. Technical Characteristics of the Designed Prototype

As a result of the present investigation, a seismic data acquisition prototype has been implemented with the following technical characteristics:

- Nodes:
  - Power supply of 5 V, obtained from two power banks.
  - Microcontroller: LaunchPad CC3200 [39].
  - Serial CLI interface at 115,200 bauds for monitoring and provisioning tasks.
  - Connected to one vertical sensor (with the possibility to also connect to three-component sensors)
  - Consumption:
    ▪ 70 mA Mode Standby
    ▪ 80 mA Mode Sampling
    ▪ 140 mA pp on start.

- Raspberry Pi server:
  - Power supply through a power bank USB or USB laptop connector.
  - Model used: Raspberry Pi 4 Model B 4 GB memory [40].
  - Installed services: SSH (Secure shell), NTP server, NTP Client, SCP, Java, node.js.
  - Consumption:
    ▪ 320 mA normal operation
    ▪ 450 mA pp on start.

Two external batteries are used to power the nodes. The first battery is responsible for supplying the necessary voltage and current for the conditioning circuit and the analog-to-digital converter. The second battery is responsible for powering the CC3200 microcontroller. Both batteries have been chosen with identical characteristics: 5 V, 1 A, and a capacity of 4000 mAh. In this way, as they are powered independently, possible interferences are avoided in the case of current demand peaks that could occur in the transmission via Wi-Fi by the microcontroller.

With this power supply, the developed system can register continuously for up to 32 h without interruption. In any case, for longer recording periods, it would be enough to choose higher-capacity batteries.

Regarding the RPI server, it could be supplied power through the USB connector of the laptop (in the case of short-term measures) or by a 5 V portable battery.

**Figure 8:**
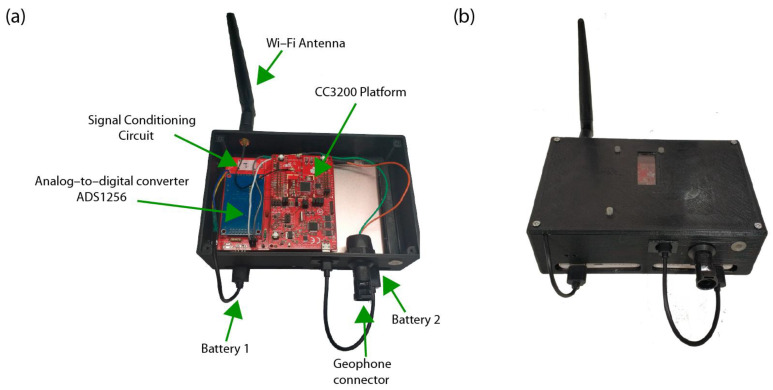
(**a**) Component distribution inside the enclosure (**b**) System enclosure.

In Figure 8, the system enclosure and the different parts inside it are shown.

The cost of each part of the installed system is detailed below:

- Nodes:
  - LaunchPad CC3200: 55.44 EUR.
  - Conditioning circuit: 45.51 EUR.
  - ADS1256 module: 10.25 EUR.
  - Two Power Bank 4000 mAh: 23.76 EUR.

- RPI server:
  - Raspberry Pi Kit: 101.33 EUR.
  - Antenna BrosTrend AC650: 19.99EUR.

For the experimentation part, six nodes and an RPI server have been used with a total cost of 931.08 EUR.

### 3.2. Test on Digitizer

#### 3.2.1. Synchronization Test

The first test carried out tries to demonstrate the correct synchronization of the signals recorded by all the nodes. For this experiment, a signal generator (Multicomp MP750064) has been connected directly to the ADS1256 module of each of the six analyzed nodes. Thus, the six nodes receive the same input sinusoidal signal of 1 Hz, 2.4 V_pp_, and an offset of 2.4 V.

**Figure 9:**
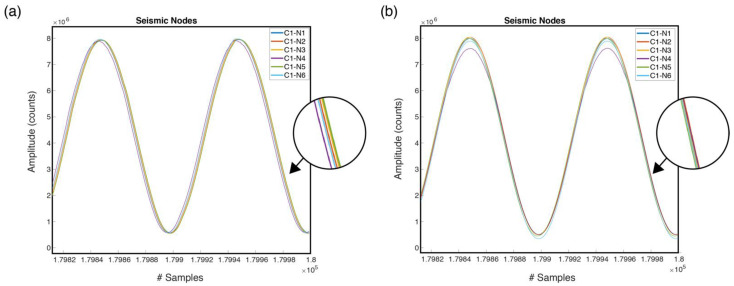
End of a 30 min register with the data obtained for the 6 nodes: (**a**) with Timer_A0 configured with the default value. (**b**) with Timer_A0 configured with the calibrated value.

Taking into account the frequency of the internal clock of the microcontroller, i.e., 80 MHz, and considering a sampling frequency of 100 Hz, the Timer_A0 counter must be set to the default value of 800,000 counts. However, even if the six nodes are completely synchronized, there is always time drift of each node, and as time goes by, they will become more and more out of phase. For example, in Figure 9a is shown the end of one 30 min register, where a small deviation between nodes is observed. For this reason, it is advisable to perform a node calibration prior to measurements, which will indicate the precise value to configure in the Timer_A0 register. This value will be saved in the internal flash memory of the node and will be used every time that the node is switched on. Figure 9b shows the end of a 30 min record (180,000 samples) with the calibration value set to Timer_A0. In this case, it is observed that there is no deviation in the signal sampled from the nodes.

**Figure 10:**
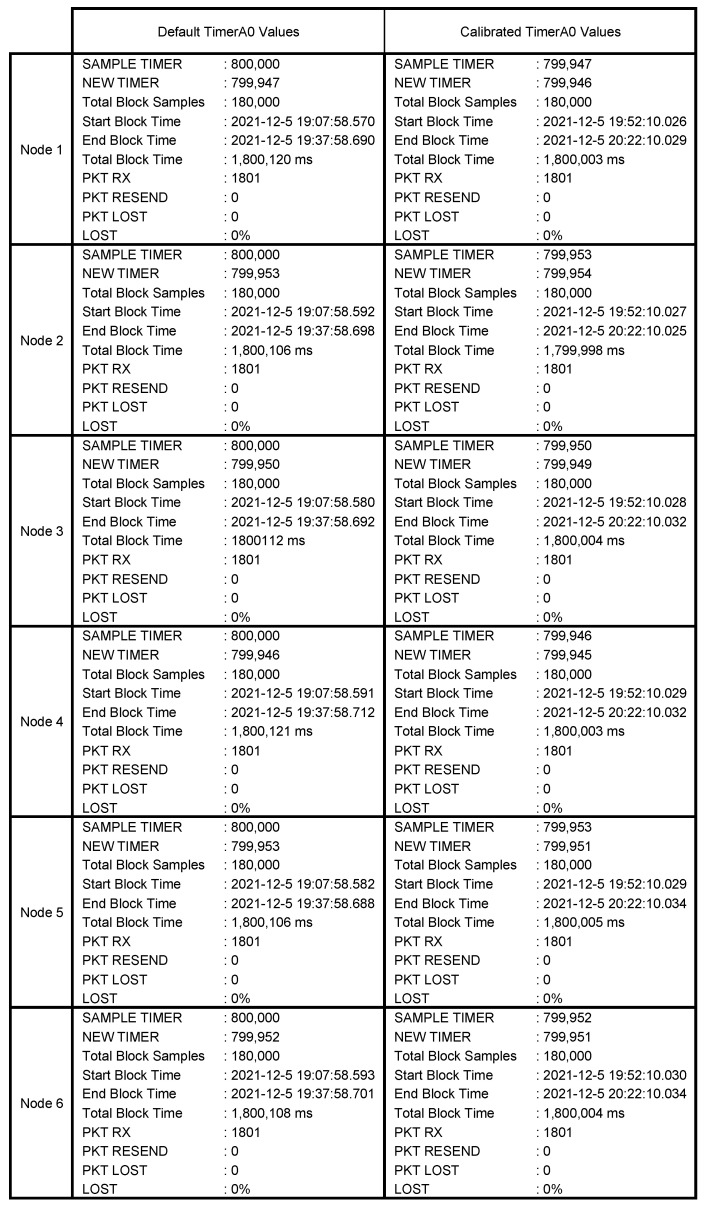
Results of the 30 min register for the six nodes.

The results of the two registers are shown in Figure 10, where it can be seen in the second column how the duration times of the registers (Total Block Time) of each node have a smaller deviation, just a few milliseconds. For example, node 1 took a total of 1800.120 s to register 30 min with the default Timer_A0 of 800,000. However, in the second register, with a Timer_A0 value of 799,947, it took a total of 1800.003 s. In the second register, it can be stated that the sampling frequency is exactly 100 Hz.

For records longer than 30 min, even if the values given by the calibration have been configured, deviations will continue to occur that will increase over time. To minimize this problem, the Synchro option should be activated, which will perform an auto-tuning of each node at the moment that the mean of the time difference is greater than 10 milliseconds while it is being recorded [30] (p. 21).

#### 3.2.2. Experimental Sensitivity

In order to check the sensitivity of the developed system, two continuous signals of 2 and 4 V, covering the dynamic range of the ADC (0–5 V), have been applied to the ADS1256 module of the six nodes. The output voltage was recorded for 300 s at the sampling frequency of 100 Hz. Thus, the sensitivity was computed as the average number of digital counts measured on the recordings.

The average value of each series of records and the corresponding number of counts have been calculated and compared with the theoretical value for the six nodes (Table 2).

The obtained results show that: (i) The continuous voltage does not affect the sensitivity, which remains with small deviations for all the cases; (ii) the six nodes have very similar sensitivity; (iii) the theoretical number of counts is a bit lower than the obtained experimental values, with an error smaller than 0.8% of the sensitivity for all the analyzed cases. In the Instrument Workshop: Site Effects Assessment Using Ambient Excitations [53], 12 commercial digitizers were tested, and the estimated errors were between 0.03% and 7.71%. Therefore, it can be said that the developed system is within the standards of commercial equipment.

#### 3.2.3. Internal Noise Stability

In this test, the internal noise characteristics and the deviation from the reference voltage are analyzed. For them, the inputs of each node have been short-circuited, and six records of 5 min have been created. In Table 3, the obtained results are shown.

It can be observed that the voltage measured with the short-circuited inputs is very close to the reference voltage configured at the input of the INA128 amplifier, which is 2.5 V (i.e., offset). The maximum average deviation with respect to the reference voltage is only 0.04%, which corresponds to node 3.

With respect to the standard deviation of the internal noise, it should be highlighted that it is very small, hence its values are expressed in picovolts. The nodes that present a greater deviation are 4 and 6, which maybe is due to the fact that they registered some peaks of voltage during the recordings. Even in the worst case, the standard deviation hardly reaches 2.6 nV, which gives insights into the low-noise characteristic of the designed conditioning circuit.

#### 3.2.4. Array Coverage

Several measurements have been carried out to determine the maximum coverage of the Wi-Fi signal and thus obtain the maximum possible aperture of the array. The tests consisted of taking measurements using circular arrays of different radii and with six sensors uniformly distributed. Once each record was finished, the JavaScript server showed the details of the communication, including the frames forwarded by each node (Table 4). The nodes that are not indicated in Table 4 are because they did not forward any frame.

In the tests carried out, it has been observed that there are no forwarded frames up to a radius of 20 m. Between 30 m and 50 m, the forwarded frames are residual. Additionally, only from 100 m, a considerable increase in forwarded frames is observed.

The algorithm designed for the control and recovery of lost frames has allowed for forwarding them at later intervals, avoiding any loss of frames. Thus, thanks to the developed algorithm, the array coverage has been considerably increased, covering an aperture of up to 250 m for circular array deployments.

### 3.3. Field Experiment

To test the suitability of the developed acquisition system for recording seismic noise array measurements, we have carried out several field campaigns along the province of Alicante (southeast Spain), and the results of the corresponding analysis have been compared with results obtained using a commercial digitizer (24-channel P.A.S.I. equipment). Concretely, the sites selected for the field campaigns have been Almoradí (0°46′59.17″ W, 38°6′42.14″ N), Catral (0°48′19.85″ W, 38°9′26.09″ N), Dolores (0°44′38.28″ W, 38°8′39.79″ N), and Rojales (0°43′8.00″ W, 38°5′25.97″ N). The measures were taken between March and May 2022 during calm weather in order to avoid possible wind-induced disturbances at low frequencies.

**Figure 11:**
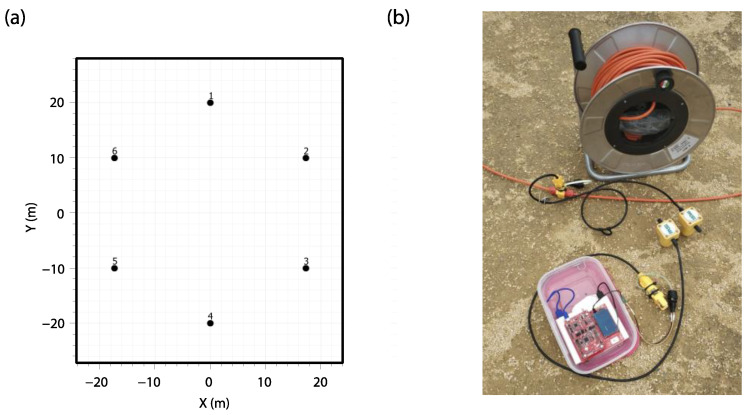
(**a**) Array geometry (**b**) One measurement point with one geophone (right one) connected to the P.A.S.I. seismic cable and the other geophone (left one) connected to one of the nodes.

In each of the sites, a circular layout of 40 m in diameter was deployed with six measurement points distributed around the circumference (Figure 11a). A vertical 4.5 Hz geophone connected to the developed acquisition system was placed at each point, and a second vertical 4.5 Hz geophone connected to the P.A.S.I. equipment was placed right next to it (Figure 11b). In this way, the six nodes of our system were placed together with the six geophones of the commercial equipment in order to carry out the measurements together at the same time and allow the subsequent comparison. The 4.5 Hz geophones used have a sensitivity of 82 V/m/s.

The aperture of the array and the number of sensors determine the theoretical array response and the measurement limits in terms of the wavenumber, k. In this way, the maximum resolution of the array depends on the minimum detectable wavenumber, i.e., kmin/2, while the maximum reliable wavenumber, i.e., kmax/2, is limited by the aliasing effect. In our case, for the deployed array layout, the kmin/2 and kmax values are 0.0563 and 0.306 rad/m, respectively.

The data acquisition was configured with a sampling frequency of 100 Hz, recording for 15 min. In the case of the commercial equipment, the sampling frequency is 125 Hz.

**Figure 12:**
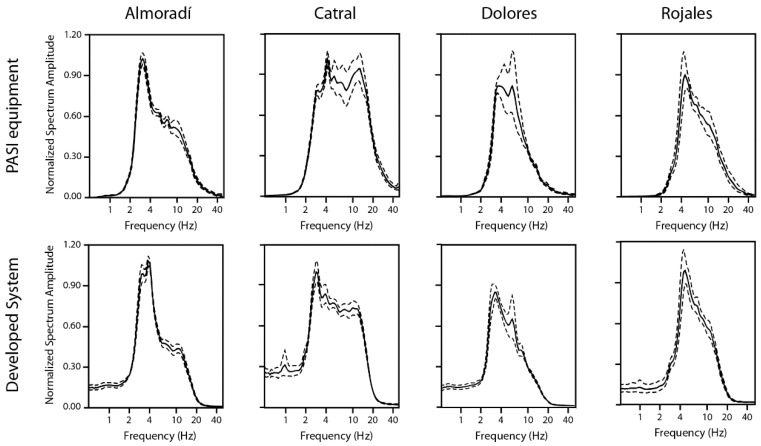
Normalized spectrum amplitude for the different analyzed sites, using P.A.S.I equipment and the developed system. The continuous black line indicates the mean value, and the dashed lines are the mean value ± standard deviation, respectively.

In order to estimate the spectrum of the recorded noise and test the suitability of the sensor-digitizer instrumental chain, we have compared the normalized spectrum of the recordings obtained with the commercial equipment (i.e., P.A.S.I.) and the developed system. In Figure 12, the obtained spectra are shown for the different analyzed sites.

These curves present different shapes and amplitudes from one site to other due to the different characteristics of the recorded noise and the soil underneath at the selected sites.

In general, the results obtained for both pieces of acquisition equipment are very similar in terms of shape, amplitude, and peaks. The major differences are observed in the Catral site for frequencies higher than 10 Hz. This is because the low-pass filter of the developed system has been configured to 15 Hz, which is enough for analyzing the frequencies of interest and to assure a sufficient drop in the Nyquist frequency.

Finally, in order to validate the suitability of the developed system for the analysis of the array measurements, the recorded signals were analyzed with the f-k method [16,17,18], which is one of the most used array techniques. As a result of the f-k analysis, the velocity of the surface waves as a function of the frequency (i.e., the dispersion curve) was estimated.

The analysis was carried out with the Geopsy software [54]. In the time domain, the recorded signals were divided into frequency-dependent time windows (which include 50 periods). Meanwhile, in the wavenumber domain, the analysis was determined by the grid step and the grid size parameters, which are directly related to the minimum and maximum wavenumber limits, i.e., the kmax/2 and kmin/2 values, respectively.

**Figure 13:**
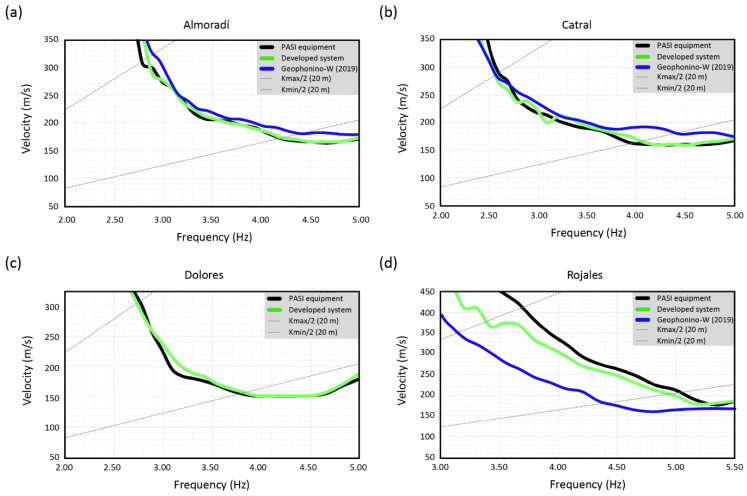
Dispersion curves estimated with the developed data acquisition system (green color), Geophonino-W [33] (blue line), and the commercial equipment (black color) for the sites under study: (**a**) Almoradí, (**b**) Catral, (**c**) Dolores, and (**d**) Rojales. Wavenumber limits (k_max_/2 and k_min_/2) are represented with dashed lines.

In Figure 13, the dispersion curves estimated for the four analyzed sites, using the commercial equipment (black line) and the developed system (green line), are shown. Besides, for comparison purposes, the dispersion curves obtained in a previous work [33] using the Geophonino-W system (blue line), are also shown for three of the four analyzed sites. In this case, the field campaigns were carried out in 2019, using ten vertical 1 Hz sensors for each array. For the present work, it was not possible to repeat the measurements with Geophonino-W [33] since we do not currently have the vertical 1 Hz sensors (they were borrowed by the Institut Cartogràfic i Geològic de Catalunya). On the other hand, the use of the 4.5 Hz geophones with the Geophonino-W system [33] was also not possible due to the low sensitivity of the sensors and the internal noise of Geophonino-W, which is twenty times higher than the internal noise of the developed system.

Anyway, the comparison is possible because the used technique is based on the assumption of a stochastic wavefield, which is stationary, both in time and space [12,55].

The intersection of the theoretical wavenumber limits and the experimental dispersion curves provides the valid frequency range for each case. As it was commented previously, the measurement limits, in terms of the wavenumber, depend on the aperture of the array and the number of sensors; i.e., the theoretical array response. Meanwhile, the frequency limits depend on the theoretical array response but also on the soil characteristics (through the experimental dispersion curve).

For the Almoradí site (Figure 13a), the valid dispersion curve comprises between 2.8 and 4.4 Hz, approximately. Both curves, the one estimated with the developed system and the one estimated with the P.A.S.I. equipment, are almost identical in this frequency range.

In the case of Geophonino-W [33], a circular array with a diameter of 40 m was deployed. The valid frequency range was established between 3 and 7 Hz. Although the diameter of the array is the same in both experiments, we can observe how, as the number of stations increases (ten sensors in the case of Geophonino-W [33]), the distance between stations decreases, and the maximum limit associated with the aliasing increases.

Therefore, the differences that could be observed at frequencies higher than the maximum frequency limit (see [33]) are due to the array layout (aperture and number of stations) and not so much to the equipment used.

In the valid frequency range associated with the developed system, the obtained curve also shows a good agreement with the one estimated by Geophonino-W in 2019 [33].

In the case of the Catral site (Figure 13b), the frequency range goes from 2.6 to 4.0 Hz, presenting a great concordance between the developed and the commercial systems in this interval. In the case of Geophonino-W [33], a circular array, with a diameter of approximately 40 m and ten 1 Hz sensors, was implemented near the study site in 2019, in a place with the same soil characteristics. The valid frequency range was estimated between 3.5 and 8.2 Hz, although above 6 Hz, the response is approximately constant.

In the common valid frequency range, the curves obtained by Geophonino-W [33] do not show significant differences with respect to the ones obtained by the developed system.

For the Dolores site (Figure 13c), the validation of the dispersion curve is limited to the interval between 2.8 and 3.8 Hz. In this frequency range, the curves obtained with the proposed and the commercial systems follow a similar behavior. In this case, the comparison with Geophonino-W is not possible [33] since array measurements were not taken for this site in 2019.

Finally, in the Rojales site (Figure 13d), the dispersion curves could be reduced to the frequency range of 3.7–5.0 Hz. This is the case where the greatest discrepancies are observed between the developed and the commercial systems, mainly at low frequencies. Moreover, the major difference in velocity is around 40 m/s at the frequency of 3.7 Hz, which is within the error or standard deviation of both curves.

For Geophonino-W [33], the array measurements were taken in another place near the study site. The available free area conditioned the site and the array layout. Thus, the ten 1 Hz sensors were distributed around an ellipsoidal-shaped geometry with a major radius of 43 m and a minor radius of 26 m. According to that, the valid frequency range was 3–8 Hz. In this case, the comparison of the Geophonino-W dispersion curve [33] with the other two shows a greater deviation than that obtained for the rest of the sites (i.e., Almoradí and Catral). These discrepancies could be due to the fact that the measurements were not taken exactly in the same place, and, therefore, the characteristics of the soil could be somewhat different. Moreover, the observed variation could be within the error ranges established by the corresponding standard deviation of the estimated curves.

In a conclusion, from the experimentally obtained results, the suitability of the developed system for seismic noise array measurements can be demonstrated. For all the analyzed sites, the estimated dispersion curves are in agreement with the ones obtained by the commercial equipment.

It is important to highlight that the system has been developed under the premises of low noise, low consumption, and low cost, allowing the use of low-sensitivity geophones. In this sense, we have obtained results comparable to those of the commercial and the Geophonino-W [33] systems simply using six 4.5 Hz geophones. In contrast, with the Geophonino-W system [33], ten 1 Hz sensors were required for obtaining similar curves. The economic savings are incredibly significant (around 10,000 EUR) since the price of the six 4.5 Hz geophones is even considerably less than the cost of one single 1 Hz sensor.

## 4. Conclusions

In this work, a wireless data acquisition system for seismic noise array measurements has been designed, implemented, and tested. The developed system uses Wi-Fi technology. The wireless characteristic allows simpler and faster deployment of the instrument in the field to perform array measurements. The recorded signals are sent to the RPI server and can be visualized in real-time, which allows for checking for the right performance of the measures directly in the field. In addition, as the signals are collected by the RPI server in real time, they are available as soon as the measurements are finished. Therefore, it is not necessary to access each node separately for retrieving the data.

A new low-noise conditioning circuit has been also developed, specifically designed for the acquisition of seismic noise. An application protocol has also been designed to transmit the samples securely with flow control and error detection. The memory of the CC3200 board has been used to the maximum to define a buffer that allows storing up to 100 s of samples, providing a security interval in case of a communications outage.

Regarding the synchronization of the nodes, two phases have been implemented for the complete synchronization of the system, ensuring a maximum deviation of 5 ms between any node and the RPI server. If this limit is reached, the node is able to adjust again automatically.

Different tests have been applied to assess the synchronization among the signals registered by the nodes and quantify the sensitivity, the internal noise, and the maximum deviation with respect to the reference voltage. The maximum array coverage has also been analyzed, obtaining an aperture of up to 250 m for circular arrays. In addition, several field campaigns have been carried out to study the performance of the developed system for the acquisition of seismic noise array measurements. The recorded measurements have been analyzed with the f-k method, and the obtained results have been compared with the ones obtained using a commercial digitizer (P.A.S.I. equipment), as well as the ones obtained by another developed system (Geophonino-W, [33]) in 2019. For the experiments carried out, the results (i.e., the dispersion curves) obtained with the recordings of both instruments are in very good concordance.

Finally, another of the objectives achieved is to have used low-cost and low-power consumption components in the developed system. In terms of software, open-source tools have also been used.

## Figures and Tables

**Table 1:** Type of messages.

Type of Messages	Type	Code
Echo Reply	0	−
Sampling Order, START immediately	1	1
Sampling Order, STOP	1	2
Sampling Order, START without sending	1	3
Sampling Order, START in next second	1	5
Samples Reply	2	1
ACK Samples Reply	2	2
Hello	5	−
Set Timer, set Sampling Timer counter value	6	1
Set Timer, ACK	6	2
Set Timer, set Delay Timer value in milliseconds	6	5
Set Timer, ACK Delay Timer value in milliseconds	6	6
TimeStamp, initial sampling	7	1
TimeStamp, Stop-and-Go	7	2
TimeStamp, finish sampling	7	3
Echo Request	8	−
SYNC CLK Request	9	1
SYNC CLK Reply	9	2
SYNC CLK Return values	9	3

**Table 2:** Experimental sensitivity results for the six nodes.

Input Continuous Voltage (V)	2	4
Sampling rate (Hz)	100	100
Dynamic range (bits)	23	23
Theoretical value (counts)	3,355,443.2	6,710,886.4
node 1 channel (counts)	3,378,909.3	6,729,637.0
N1 deviation from theoretical value (%)	0.69%	0.28%
node 2 channel (counts)	3,381,885.8	6,735,540.9
N2 deviation from theoretical value (%)	0.78%	0.37%
node 3 channel (counts)	3,378,067.6	6,727,741.5
N3 deviation from theoretical value (%)	0.67%	0.25%
node 4 channel (counts)	3,378,033.3	6,727,897.9
N4 deviation from theoretical value (%)	0.67%	0.25%
node 5 channel (counts)	3,378,449.0	6,728,754.4
N5 deviation from theoretical value (%)	0.68%	0.27%
node 6 channel (counts)	3,359,006.8	6,690,098.8
N6 deviation from theoretical value (%)	0.11%	0.31%

**Table 3:** Internal noise and deviation from the reference voltage.

	N1	N2	N3	N4	N5	N6
Sampling rate (Hz)	100	100	100	100	100	100
Dynamic range (bits)	23	23	23	23	23	23
Average (counts)	4,308,888	4,322,369	4,364,737	4,321,730	4,176,427	4,312,831
Average (V)	2.568	2.576	2.601	2.575	2.489	2.570
Standard deviation (pV)	414	535	548	1565	585	2638
Deviation from Vref (2.5 V)	0.02%	0.03%	0.04%	0.03%	0.004%	0.02%

**Table 4:** Frames forwarded for different array apertures.

Radius	Frames Forwarded by Each Node	Total Frames Forwarded
20 m	–––	0
30 m	N2 = 2, N5 = 2	4
40 m	N4 = 4	4
50 m	N2 = 2	2
60 m	N2 = 4, N4 = 2, N5 = 2, N6 = 2	10
75 m	N1 = 12, N2 = 4, N3 = 2, N4 = 4, N5 = 2, N6 = 48	72
87 m	N1 = 8, N2 = 6, N3 = 8	22
105 m	N1 = 13, N2 = 10, N3 = 300, N4 = 47, N5 = 57, N6 = 4	431
125 m	N1 = 6, N2 = 116, N3 = 145, N4 = 353, N5 = 10, N6 = 40	670

## Data Availability

All data included in the manuscript are available upon request by contacting with the corresponding author.

## Abbreviations

It describes the abbreviations used throughout this paper.

**Abbreviations**	**Description**
SNR	Signal noise ratio
UDP	User datagram protocol
TCP	Transmission control protocol
RPI	Raspberry Pi
SCP	Secure copy protocol
NTP	Network time protocol
CLI	Command line interface
UART	Universal asynchronous receiver-transmitter
ADC	Analog-to-digital converter
WAP or AP	Wireless access point
UTC	Universal time coordinated
mTS	Milliseconds time stamp
TS	Time stamp
PTP	Precision time protocol
SSH	Secure shell
PCB	Printed circuit board
SPI	Serial peripheral interface
